# Learning from a cluster randomized controlled trial to improve healthcare workers’ access to prevention and care for tuberculosis and HIV in Free State, South Africa: the pivotal role of information systems

**DOI:** 10.3402/gha.v9.30528

**Published:** 2016-06-23

**Authors:** Annalee Yassi, Prince A. Adu, Letshego Nophale, Muzimkhulu Zungu

**Affiliations:** 1 Global Health Research Program, The University of British Columbia (UBC), Vancouver, BC, Canada; 2 Provincial Occupational Health Unit, Free State Department of Health, University of the Free State, Bloemfontein, South Africa; 3 National Institute for Occupational Health, A Division of the National Health Laboratory Service, Johannesburg, South Africa; 4 School of Health Systems and Public Health, University of Pretoria, Pretoria, South Africa

**Keywords:** cluster randomized controlled trial, information systems, tuberculosis, HIV, healthcare workers

## Abstract

**Background:**

Occupational tuberculosis (TB) continues to plague the healthcare workforce in South Africa. A 2-year cluster randomized controlled trial was therefore launched in 27 public hospitals in Free State province, to better understand how a combined workforce and workplace program can improve health of the healthcare workforce.

**Objective:**

This mid-term evaluation aimed to analyze how well the intervention was being implemented, seek evidence of impact or harm, and draw lessons.

**Methods:**

Both intervention and comparison sites had been instructed to conduct bi-annual and issue-based infection control assessments (when healthcare workers [HCW] are diagnosed with TB) and offer HCWs confidential TB and HIV counseling and testing, TB treatment and prophylaxis for HIV-positive HCWs. Intervention sites were additionally instructed to conduct quarterly workplace assessments, and also offer HCWs HIV treatment at their occupational health units (OHUs). Trends in HCW mortality, sick-time, and turnover rates (2005–2014) were analyzed from the personnel salary database (‘PERSAL’). Data submitted by the OHUs were also analyzed. Open-ended questionnaires were then distributed to OHU HCWs and in-depth interviews conducted at 17 of the sites to investigate challenges encountered.

**Results:**

OHUs reported identifying and treating 23 new HCW cases of TB amongst the 1,372 workers who used the OHU for HIV and/or TB services; 39 new cases of HIV were also identified and 108 known-HIV-positive HCWs serviced. Although intervention-site workforces used these services significantly more than comparison-site healthcare staff (*p*<0.001), the data recorded were incomplete for both the intervention and comparison OHUs. An overall significant decline in mortality and turnover rates was documented over this period, but no significant differences between intervention and comparison sites; sick-time data proved unreliable. Severe OHU workload as well as residual confidentiality concerns prevented the proper implementation of protocols, especially workplace assessments and data recording. Particularly, the failure to implement computerized data collection required OHU staff to duplicate their operational data collection duties by also entering research paper forms. The study was therefore halted pending the implementation of a computerized system.

**Conclusions:**

The significant differences in OHU use documented cannot be attributable to the intervention due to incomplete data reporting; unreliable sick-time data further precluded ascertaining the benefit potentially attributable to the intervention. Computerized data collection is essential to facilitate operational monitoring while conducting real-world intervention research. The digital divide still requires the attention of researchers along with overall infrastructural constraints.

## Introduction

Fuller and Potvin (1) argue that understanding the impact of social context on population health interventions should be a key research objective. Reynolds et al. (2) further emphasize the importance of identifying the circumstances under which health service intervention studies could be useful. Adding to growing scholarship on research methods for evaluating complex interventions (3–11), Reynolds focused attention on ‘data collection tools that form the fabric of the evaluation components of trials’. He argued that, to inform implementation and scale-up, studies need to reflect on the real life setting of the trial, and insisted that complex interventions in low-income country settings entail challenges that could not easily be predicted. In 2014, we outlined the steps we took in launching a large collaborative randomized controlled trial (RCT) in Free State, South Africa, to better understand the determinants of successful implementation of the World Health Organization (WHO)-International Labour Office (ILO)-UNAIDS guidelines on improving access for healthcare workers (HCWs) to prevention and care for tuberculosis (TB) and HIV (12). We argued that such studies need to be seen as iterative, rather than having distinct start and end dates, precisely because, as Reynolds et al. observed, new issues always arise. The present article updates this discussion, presenting findings after the 1-year review that led us to prematurely end the operational stage of this RCT and attend to the major barriers encountered. Like Reynolds et al., we believe that identifying the real-world challenges that emerge are important findings worth sharing, and like Fuller and Potvin, our experience re-emphasizes the importance of the social context.

**Fig. 1:**
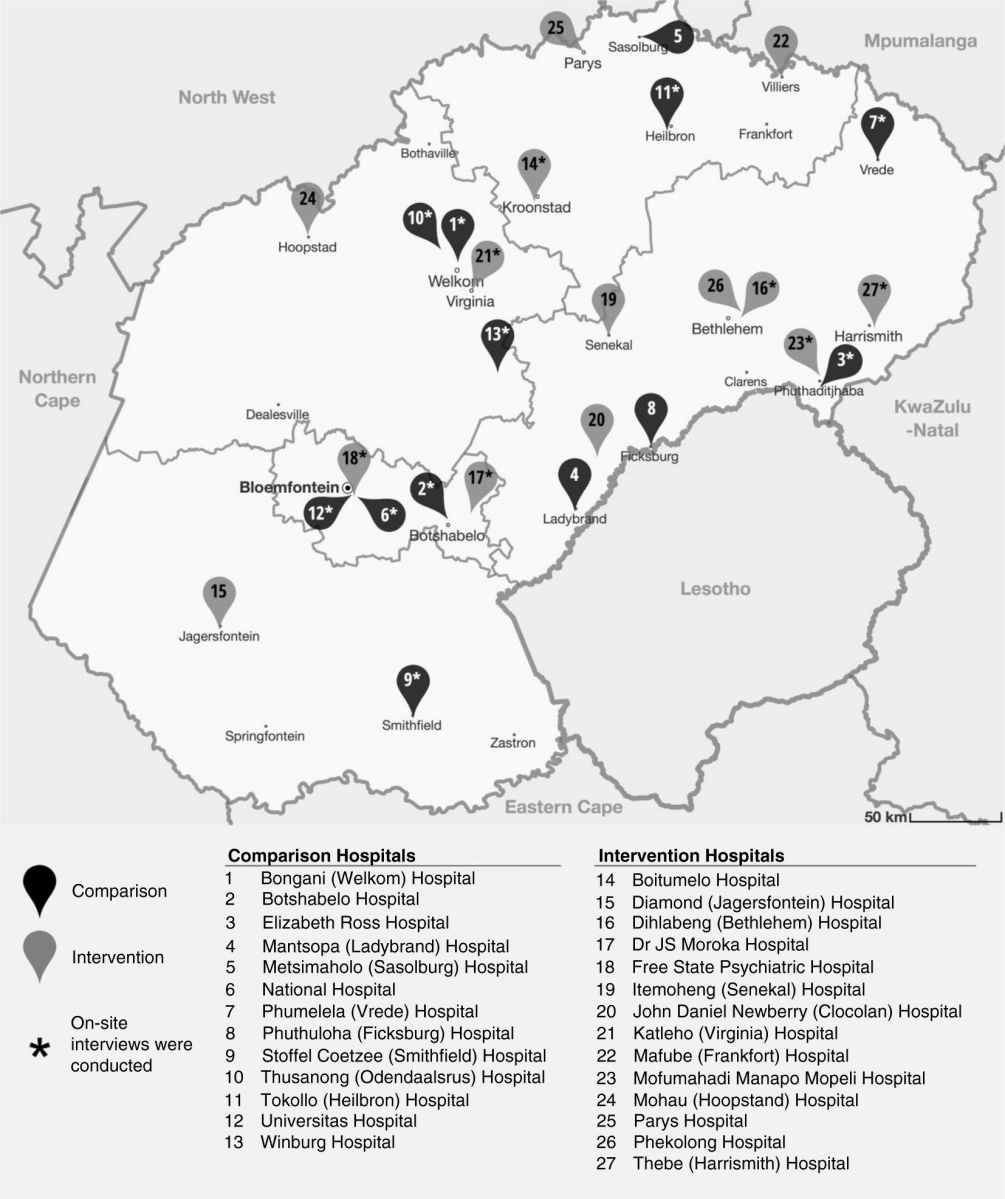
Map of Free State Province showing study sites.

As described previously (12), after almost 7 years of international relationship-building and preliminary research, a 2-year pragmatic, cluster RCT was launched in July 2013 at 27 hospitals in the Free State province, South Africa (See Fig. 1). The purpose was to ascertain whether, and under what conditions, a combined workforce and workplace intervention can improve health outcomes and strengthen occupational health and infection control services. The study aimed to ascertain whether such an intervention could: 1) improve the health outcomes of the healthcare workforce, as defined by the incidence of TB disease and overall mortality, and sick-time and turnover rates; 2) increase clinical service uptake at the occupational health units (OHUs), as determined by staff usage of services for TB and HIV; and 3) strengthen occupational health and TB infection control practices, as assessed by the frequency, findings, and actions from workplace assessments for TB infection control. These assessments or workplace audits were to be routinely conducted, according to national and international norms, as well as following any time a HCW contracts TB, which were referred to as ‘issue-based’. Extensive planning, pilot work, and feasibility testing had been conducted (12–16) by a joint team of international researchers, national experts, and provincial personnel, with many meetings, as well as large training sessions. The RCT protocol included conducting a review after the first year of data collection, so that needed changes could be implemented.

The purpose of this article is to present the synthesized findings of the mid-term evaluation. In so doing, we draw attention to a decision we explained previously (12) regarding use of the data collection system we designed, the Occupational Health and Safety Information System or ‘OHASIS’, composed of modules to record worker health, track workplace injuries or incidents, facilitate investigations, manage waste and produce relevant reports, and so on (17). A special HIV and TB module was designed to record clinical data for daily service use, not just for research purposes. We noted ‘… [T]he team continues to seek the most convenient way for busy occupational health practitioners to systematically collect the data needed for operational purposes, as well as for our RCT, without adding burden. With slow internet connectivity speeds, poor computer access, and limited computer literacy, this is an ongoing challenge. …’. We eventually decided not to use OHASIS on-site, but rather collect relevant indicators at the OHU on paper and have these data faxed to the local university to enter the data electronically. As will be shown in this article, we regret launching this RCT using paper-based forms, and share this lesson, as well as some others, so that other researchers can learn from this and other decisions we regretted.

## Methods

We randomized 27 public hospitals in the Free State province into two arms: 13 intervention hospitals and 14 comparison sites. Inclusion criteria included having an on-site OHU with trained staff; OHUs based off-site or without designated staff were excluded. Hospitals of similar size (based on numbers of staff) and in the same district, if possible, were deliberately paired (there was one group of three hospitals) to ensure the equal distribution of large and small hospitals across the different districts in both intervention and comparison arms. A coin toss was used to randomly allocate one of each pair into the intervention arm. Both intervention and comparison sites were instructed to conduct bi-annual and issue-based workplace infection control assessments; they were also instructed to offer their workforce confidential TB and HIV counseling and testing as well as TB treatment and prophylaxis for HIV-positive staff. Intervention sites were, in addition, instructed to conduct quarterly workplace assessments and seek assistance from national experts to implement recommended infection control practices, as well as to offer their workforce HIV treatment at the OHU. The trial was launched in July 2013, with OHUs instructed to fax their data monthly to the local university where the data was entered into OHASIS.

For the mid-term assessment, first we analyzed the mortality, sick-time, and turnover data collected in the Personnel Salaries (PERSAL) database, the human resource database used for payroll and related administrative functions within the public sector in South Africa (18, 19). Turnover was calculated as the number of employees who left during a particular year, divided by the average number of employees that worked during that year. To analyze trends in mortality, sick-time, and turnover rates, we fitted least-squares regression models to the data and compared the trend lines of the intervention and comparison groups.

For clinical HIV and TB services, as well as the assessment of the quality of workplace TB infection control audits, we assessed the OHASIS data sent by the OHUs. In keeping with ethical principles of global health research (20–22), as soon as the analysis was conducted, the data from each site were sent back to each individual OHU to share results at the earliest possible stage so that action could be taken. We then proceeded with the qualitative study to contextualize the data, distributing questionnaires to the occupational health nurses (OHN), at both intervention and comparison sites, and following up with face-to-face semi-structured interviews in 17 (10 comparison and 7 intervention sites) of the 27 OHUs. As part of the OHN interviews, the researchers explicitly inquired about the completeness of the submitted data regarding the HIV and TB services actually performed. The interviews were recorded, transcribed, and analyzed thematically, using both deductive and inductive approaches. These approaches allowed us to explore areas we preconceived were problematic, but also forced us to be open to the new information that emerged. We were specifically interested in comparing what was planned to what actually occurred, and elucidating the constraints to implementation. Following the OHN interviews, a meeting was held with other local stakeholders to discuss our observations.

Permission to proceed with the in-depth qualitative analysis was re-obtained from the Head of the Free State Department of Health; the interview guide was approved by the Research Ethics Boards of both the University of British Columbia and University of the Free State.

## Results

### Impact on mortality, sick-time, and turnover rates

**Fig. 2:**
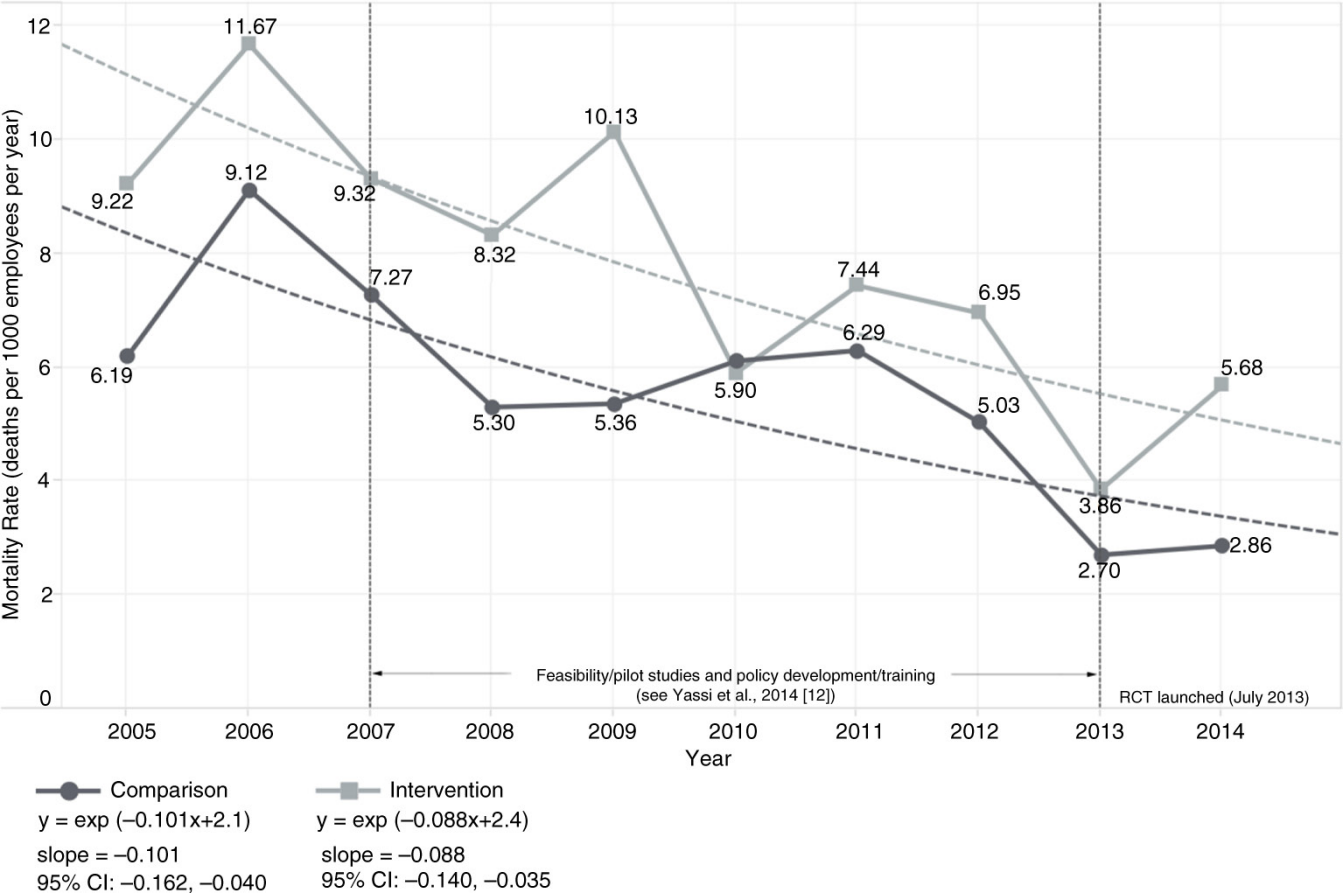
Trend in mortality rate (per 1,000 employees per year) stratified by intervention versus comparison group, 2005–2014.

**Fig. 3:**
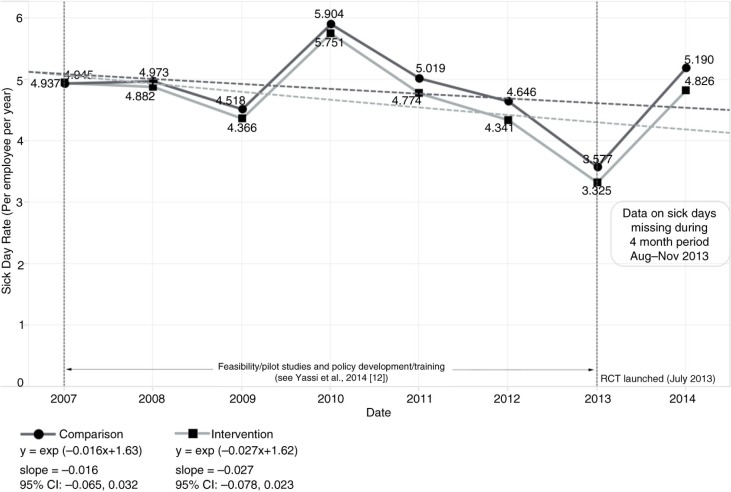
Trend in sick-time per employee per year stratified by intervention versus comparison group, 2005–2014.

**Fig. 4:**
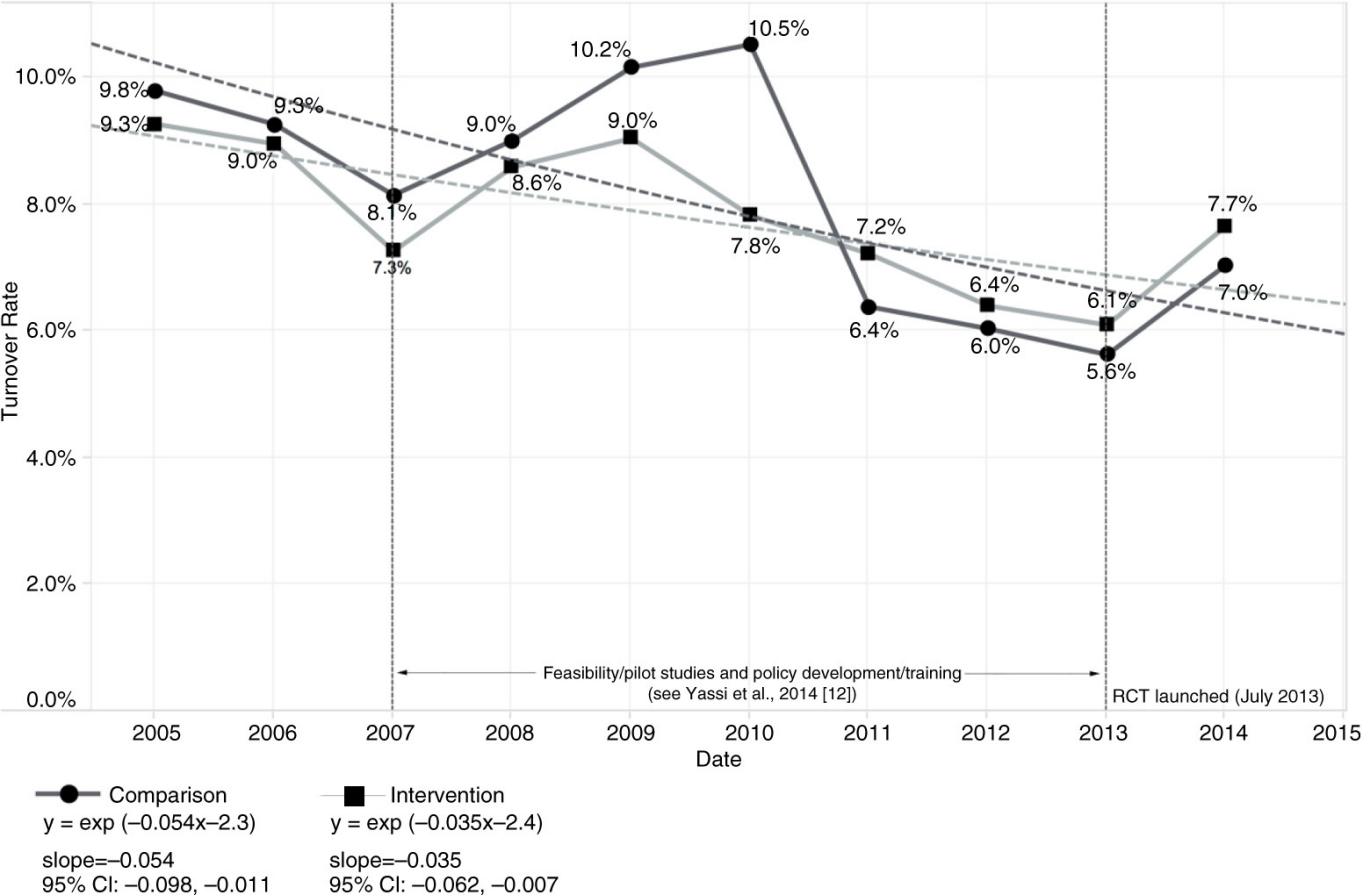
Trend in turnover rate stratified by intervention versus comparison group, 2005–2014.

The baseline human resource data consisted of 15,625 HCWs employed during the 2005–2014 period, with an average annual full-time equivalent (FTE) of 8,714 workers overall. As shown in Fig. 2, there was a significant decline in the mortality rate for both intervention [slope=−0.088, 95% CI: −0.140, −0.035] and comparison sites [slope=−0.101, 95% CI: −0.162, −0.040]. However, there was no significant difference between the intervention and comparison group trends as depicted in the overlapping confidence intervals. Missing data related to sick-time rendered any statistical analysis of this indicator unreliable (Fig. 3). Specifically, we ascertained in our interviews that, for 4 months, the Free State Department of Health had no information technology (IT) support, and data related to sick-time during this period were not properly stored; this likely at least partly accounted for the huge drop in sick-time shown in Fig. 3. Although turnover rates declined significantly, again, there was no clear difference between intervention [slope=−0.035, 95% CI: −0.062, −0.007] and comparison groups [slope=−0.054, 95% CI: −0.098, −0.011] (Fig. 4).

### TB and HIV clinical service use at the OHUs

**Fig. 5:**
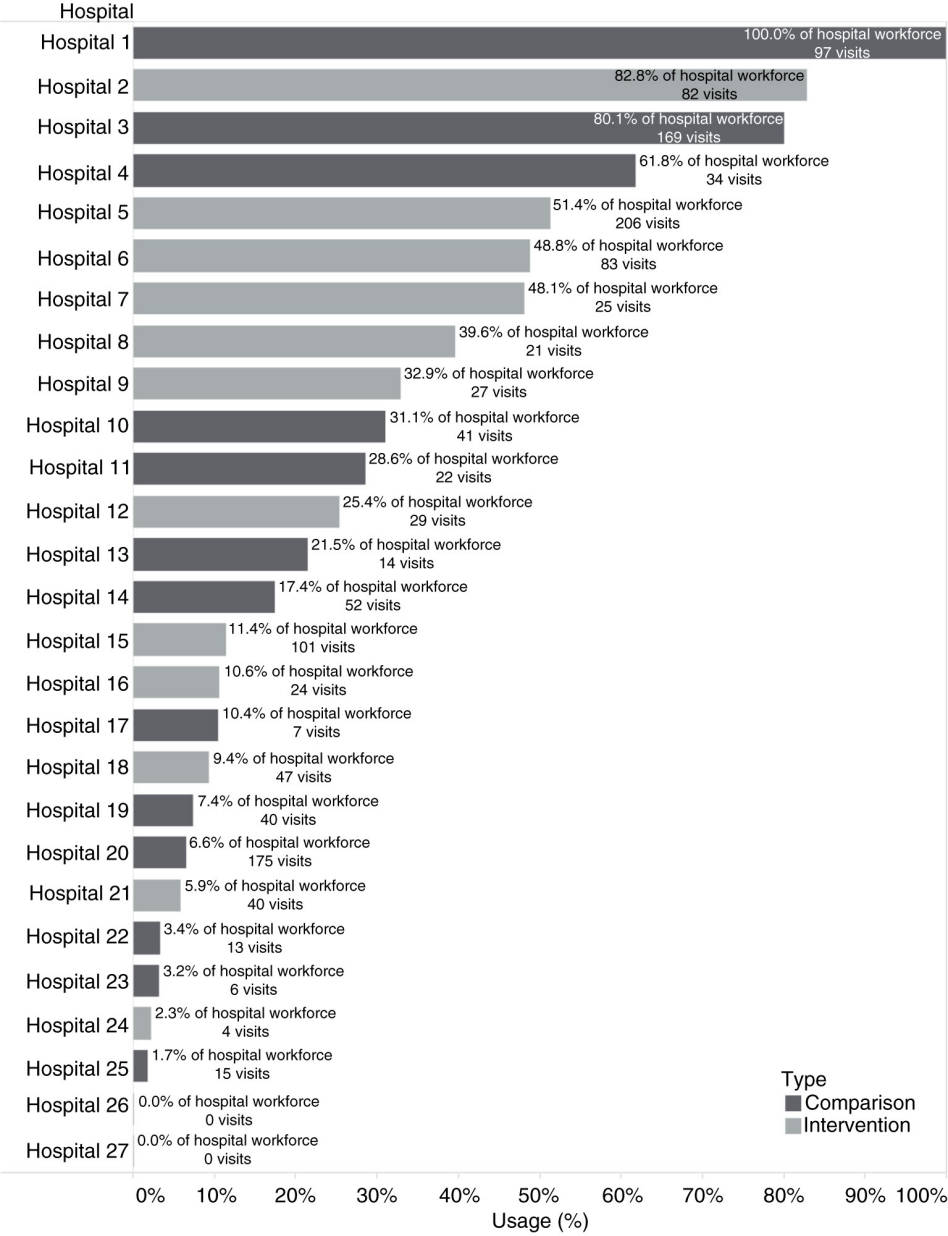
Percent of hospital workforce that accessed HIV and/or tuberculosis services in OHUs in intervention and comparison Sites, July 2013–June 2014.

**Fig. 6:**
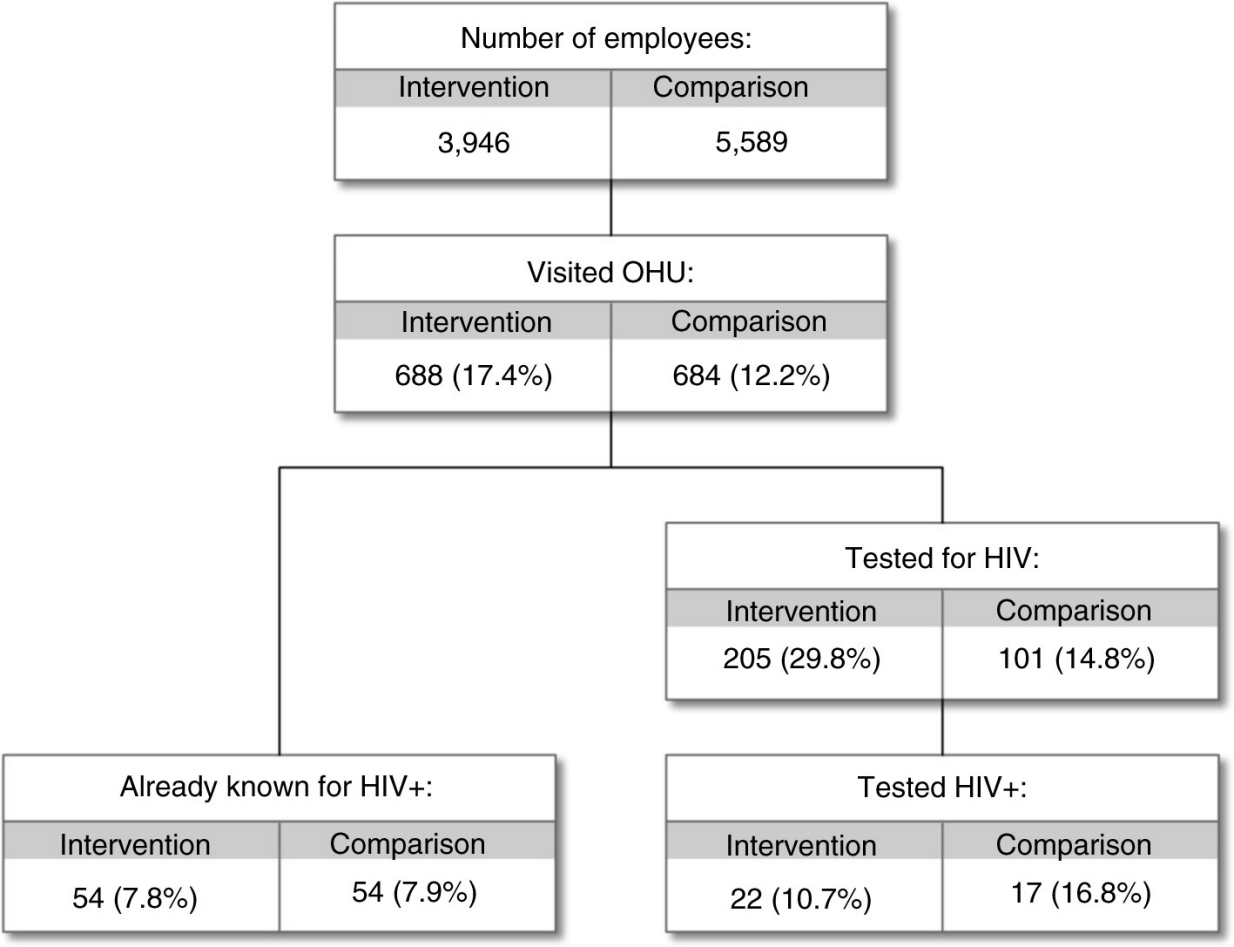
HIV counseling and testing services offered to healthcare workers in intervention and comparison hospitals, July 2013–June 2014.

**Fig. 7:**
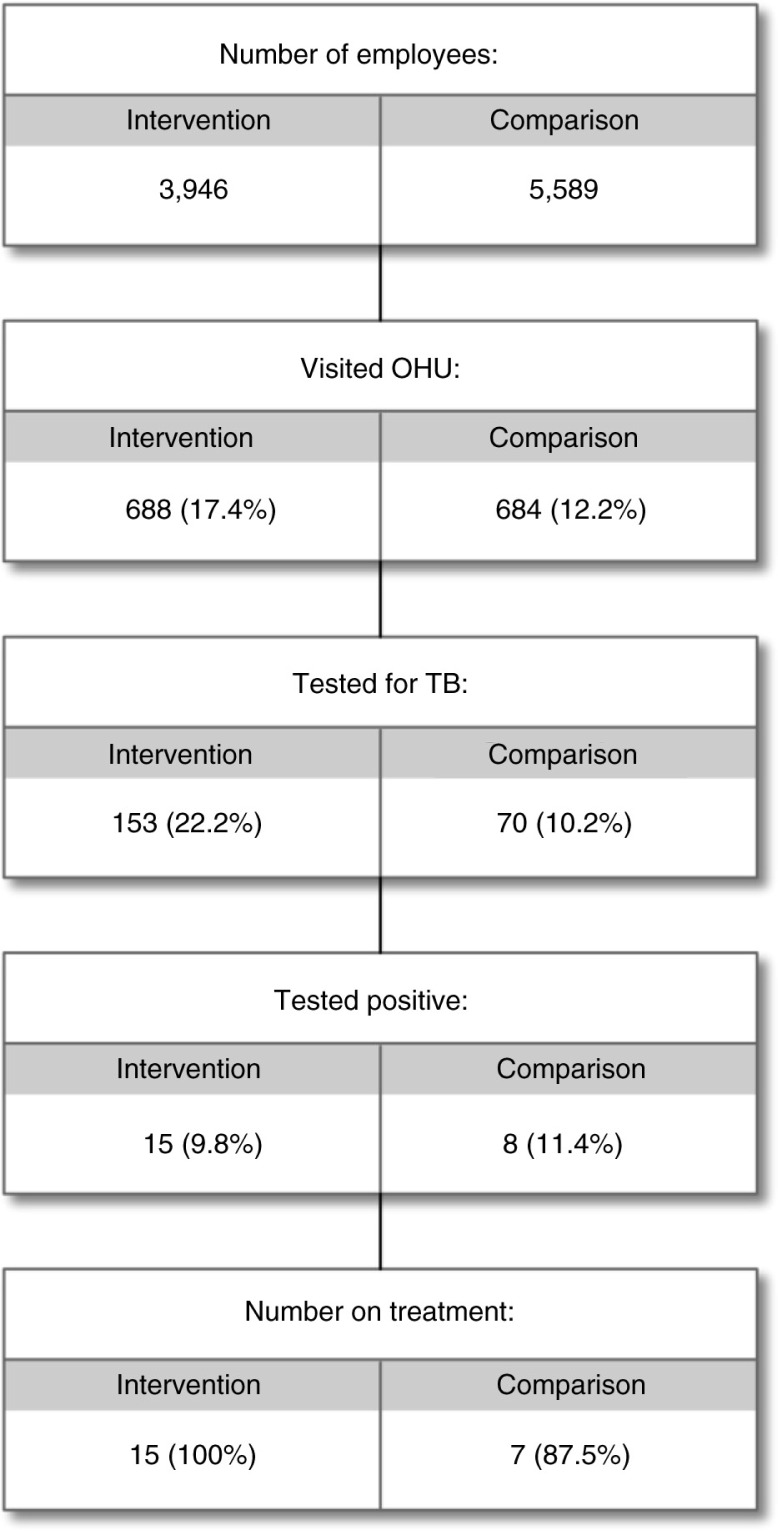
Summary of services for tuberculosis offered to healthcare workers in intervention and comparison hospitals, July 2013–June 2014.

Figures (5–7) present the clinical and clinical service data collected in OHASIS. There were a total of 3,621 OHU visits (by 1,372 health workers) from July 2013 to June 2014 for HIV and/or TB-related matters, with significantly higher average OH usage at the intervention sites (17.4% of the workforce) compared to non-intervention OHUs (12.2%), (χ^2^ [1, *N*=1,372)]=50.71, *p<*0.001). Of note, Fig. 5 showed that only two intervention OHUs and three comparison sites reported that more than 50% of their workforce had accessed HIV and/or TB services; two sites reported no clinical visit data at all. The fact that the clinical data submitted by some sites were inconsistent with what team members knew about those sites (i.e. that those OHUs were indeed providing considerable HIV and TB services) suggested that the data were incomplete and that this needed to be explored qualitatively. Nonetheless, it is noteworthy that reports documented 23 new cases of TB diagnosed; as well as 39 new cases of HIV identified and 108 previously known HIV-positive HCWs serviced at the OHUs (Figs. 6 and 7 respectively).

The qualitative study revealed (Table 1) that the clinical HIV and TB service provision, supported by regular visits and ongoing support by the clinician- researcher seconded for this study, was well executed, and OHNs were confident in their ability to perform their clinical duties. Much was learned, however, about the challenges they faced. Two main themes strongly emerged, the first related to workforce concerns about confidentiality, while the second related to the time needed to complete data recording duties.

**Table 1:** Outcome of qualitative evaluation during the mid-term evaluation

What was planned	What was found during the mid-term evaluation
1. Routine and issue-based **workplace assessments (WPA)** will be conducted in all facilities according to standard provincial and national policies.	WPAs of infection control were not done as planned. The issue-based TB infection control assessments were more commonly conducted than routine WPAs. ‘Staffing challenges’ was the most commonly cited reason. An OHN noted: *‘*We didn't do the WPA due to workload. We have other responsibilities. I am the only professional nurse working in the hospital’. Operational challenges in the hospitals were also encountered An OHN explained, ‘Right now, I have a TB patient but the patient keeps being moved from place to place (ward to ward) so it's hard to do the workplace assessment since the patient is too mobile’.

2. Nurses and doctors in intervention sites will be **supported clinically** by regular site visits by one of the researchers who is a specialist family physician employed by the Department of Health to support the ART program.	This clinical component of the protocol was executed with minimal concerns. Most OHNs found the clinical training and support they had received to be the most beneficial part of the effort.

3. In order to effect **policy and internal regulatory changes** to implement the provision of clinical care in OHUs, the research team will actively engage managers and staff from OH units, hospital executives, district, and provincial officials to contribute to the development of new policies and tools.	Although preparing the study did indeed entail formulating policies and procedures, OHNs did not concur that the study led to changes in policies or Standard Operating Procedures (SOPS) at their hospitals. An OHN from a comparison site explained why this was the case: ‘I don't think there was much buy-in from the hospital management’.

4. Anonymized individual **data** will be collected by OHU nurses to document the number of visits to the OHU for TB and/or HIV testing and treatment, the number of HCWs diagnosed at OHU with TB and/or HIV, and the outcomes of TB and/or HIV treatment. This data will be collected by OHU nurses and entered onto a database monthly by research staff at the local university using study numbers, which can later be confidentially linked to data in the human resource database (PERSAL).	The data recorded for the study represented only a fraction of the visits that actually occurred. This was the case even for OHUs that did submit considerable data for entry into the specially -designed OHASIS database. An OHN at one of the sites reported, ‘I think the data we sent reflects about 10–20% of the real picture’. The most common reason cited was that completing paper-based forms and faxing these into the local university was problematic. In most facilities, either the fax machine was broken or not available to OHU staff. None of the OHU visited had its own fax machine; most OHUs had to use the main hospital's busy fax machines. One OHN stated: ‘The forms that were supposed to be faxed, it was not easy to do that because the fax in our institution is always out of order’.

Although confidentiality within the OHU itself had been largely addressed by the preliminary work (12), the confidentiality of laboratory test results remained problematic for some OHUs, especially given the stigma associated with TB and HIV (14). In one OHU, for example, the OHN noted that patient and HCW test results are not separated when delivered from the laboratory, allowing HCWs who do not work at the OHU to easily access the results of co-workers who attended the OHU for HIV or TB services. She explained: ‘I had a problem with the results from the lab. There is no confidentiality. Patients and HCW lab results are not separated’. The OHNs further explained that HCWs are unwilling to use the OHU due to confidentiality concerns. The issue of confidentiality of laboratory results had long been identified as needing attention, but the research team was not aware the problem had not yet been completely resolved.

With respect to the OHU data collection, ONHs interviewed cited problems with fax machines that prevented them from following protocol for sending the clinical forms to the local university for data entry. Some OHNs also cited understaffing in the hospitals, as they had to be deployed to other workstations to assist in general patient care and could not complete the forms in a timely manner. When asked to estimate the extent of the completeness of the data they submitted, some OHNs indicated that completeness ranged from 50 to 80%, but others suggested that they sent data on less than 25% of the total OHU visits for HIV and TB. An important question was, ‘Had you had an electronic system to enter the data, that could generate the forms you need for your various purposes such that you would not have to complete multiple forms for the same person's visit, would you have used this system?’ The answer was 100% ‘yes’. ‘OHASIS will be very helpful. It will make it easier to record the data’, reported one OHN, a response that was widely echoed.

### How well were workplace assessments conducted?

Too few workplace assessments were recorded in OHASIS to allow an assessment of quality. Despite numerous training sessions and the collaborative development of a workplace assessment tool, not all OHNs were finding the time to conduct this task as per the study protocol. ‘We didn't do the WPA due to workload. We have other responsibilities. I am the only professional nurse working in the hospital’, explained one OHN. Some OHNs conducted workplace assessments in the follow-up to a HCW contracting TB, but not for all cases, and hardly ever as routine audits. Managers and staff from OHUs, hospital executives, and district and provincial official all approved the study and the new policies and tools. Nonetheless, adequate staffing and messaging to the OHU staff to find the time to follow the research protocols related to prevention activities still remained problematic. Despite several meetings with senior management with national and international team members, and also by the local team members between the visits of the international research team, many OHNs reported that they did not receive the necessary support from hospital managers. One OHN complained, ‘If management was involved in this research or informed, staff members would cooperate better, unlike where the OHN have to market and mobilize alone. Management was not playing any part’.

## Discussion

We chose a cluster RCT design (12) to study the impact of implementing a workplace-workforce intervention in order to respond to calls to augment the internal validity of studies (23, 24) while acknowledging the concern that RCTs are not always appropriate in low-resourced contexts (25). We argued that strong RCTs could be developed to evaluate complex population health interventions even in low and middle-income countries (LMICs), if proper attention is paid to ensuring that necessary conditions are satisfied. We also reported our collective decision ‘that funding from the research grant could support the training, monitoring and evaluation of outcomes but not additional operational staff, as the intervention would otherwise not be sustainable, even if shown to be effective’ (12). Although clinical RCTs aimed at evaluating the impact of well-defined biomedical interventions generally fund the personnel involved in data collection, our decision not to do this was pivotal in what transpired. It is always a trade-off between funding staff to ensure faithful implementation of the RCT protocol, and allowing the system to function as it would without trial funding in order to test the intervention in the real world. Our analysis of the quantitative clinical data and confirmed in the qualitative study, showed difficulties incurred at both intervention and comparison sites in implementing the intended protocols; moreover, considerable data never made it into the data system and there was no way to recover the missing data. As such, we had no choice but to end the RCT design.

We had noted in our discussions at the time of launching the study that ‘global economic factors and other policy considerations that continuously impact public sector funding, especially in LMICs (25, 26), must always be taken into account as possible factors that could undermine success of such trials’ (12). What we found in this study confirms this note of caution as, indeed, global economic and policy factors most definitely did impact the success of this trial. In South Africa, and in the Free State province in particular, there was a shortage of human resources needed to provide patient care, such that occupational health nurses and doctors were not able to fully dedicate their time to occupational health services, rendering it impossible to comply with the requirements of the study. Although the PERSAL data showed a significant decrease in mortality and turnover rates, there was no significant difference between intervention and comparison sites, with the researchers only later ascertaining that the large dip in sick-time reported in 2013 was possibly related to a loss of data due to a shortage of IT personnel. This is but one illustration of the importance of understanding real-world conditions.

The need to take measures to guard confidentiality was highlighted by the WHO-ILO in its recommendations to improve access by HCWs to HIV and TB prevention, diagnosis, treatment, care, and support (27). Numerous researchers have identified the need for strict confidentiality measures in OHUs (28–30), and indeed this is a well-established basic requirement of OHUs worldwide (31–33). We thought we had addressed this issue, and indeed we had within the OHUs themselves. However, the laboratory personnel had not been included in the pre-trial training and protocol development, which explains their less-than-perfect cooperation. Of course, there were many constraints on healthcare in Free State during that time. Cash flow was a problem, with a lack of the resources to both recruit sufficient staff and address infrastructural issues. As such, it cannot be concluded that better communication and more careful attention to confidentiality would have resolved the challenges encountered. As a discussion of the global economic forces is beyond the scope of the report, suffice it to state that the infrastructural support for this RCT was insufficient.

Our finding of the importance of a database echoes what was found by other researchers, both for research and operational purposes; for example, Reddy et al. (34) noted that the lack of a centralized database for the collection and integration of related data for easy data accessibility and analysis accounted for the limited progress in TB control. In their analysis of data quality, Dixon et al. (35) noted that resources need to be invested in building information infrastructure to address the poor data quality that exists in clinical information systems. Indeed, we were struck by the widespread agreement among our interviewees supporting the implementation of an electronic database to provide data not only for evidence-based policies but also for the day-to-day operational monitoring of workplace hazard remediation and OH service provision. On one hand, in retrospect, we feel that it was a mistake to have deferred the installation of an electronic database, because the time-consuming requirement for busy occupational health personnel to complete paper-based research forms was the Achilles heel in our plan. On the other hand, as shown by the inconsistencies in the PERSAL data, the lack of IT personnel in the Free State's Department of Health would have made this difficult. Since this analysis was completed, the Free State has strengthened its IT staffing and is now working with the National Institute for Occupational Health to solidify an implementation schedule, which will help with ultimate scale-up and sustainability (17, 36).

Finally, we note that the clinical data indicated that the OHUs identified at least 23 new cases of undiagnosed TB among HCWs. Given the problems with data recording, this can be considered a potentially large underestimate of the true problem of TB in HCWs. The percent positive among all HCWs screened was 1,676 cases per 100,000 workers, more than double the current estimate of 834 per 100,000 in the general population (37). This is consistent with the findings from many studies that estimate that HCWs have at least a two- to three-fold increased risk compared to the population they serve (38–41). Implementation of a comprehensive HIV and TB workplace policy for health workers in line with existing National policies is indeed still very much needed in South Africa, as rates of these diseases are still of extreme concern. Although we had to end this RCT prematurely, the work done before and during the trial helped raise the awareness amongst provincial health decision-makers of the need to develop and implement the needed Standard Operating Procedures in line with these policies.

## Conclusions

A greater understanding of the various challenges and constraints developed as a result of this trial, and what was learned from this final component of the study constitutes an important contribution of knowledge to decision-makers in the Free State, as well as South Africa, and beyond. As a major goal of research is to influence policy and practice, the trial can be seen as successful in this regard despite the problems encountered in the quantitative data. Indeed, there was a widespread sense that the study increased OHU utilization for TB and HIV services. It remains to be seen whether this change is sustainable and leads to an overall reduction of deaths, sick-time, and turnover rates amongst all staff at the facilities.
